# Organophosphate Insecticides Target the Serotonergic System in Developing Rat Brain Regions: Disparate Effects of Diazinon and Parathion at Doses Spanning the Threshold for Cholinesterase Inhibition

**DOI:** 10.1289/ehp.9337

**Published:** 2006-07-27

**Authors:** Theodore A. Slotkin, Charlotte A. Tate, Ian T. Ryde, Edward D. Levin, Frederic J. Seidler

**Affiliations:** 1 Department of Pharmacology and Cancer Biology and; 2 Department of Psychiatry and Behavioral Sciences, Duke University Medical Center, Durham, North Carolina, USA

**Keywords:** acetylcholine, brain development, chlorpyrifos, cholinesterase, diazinon, organophosphate insecticides, parathion, serotonin receptors, serotonin transporter

## Abstract

**Background:**

In the developing brain, serotonin (5HT) systems are among the most sensitive to disruption by organophosphates.

**Objectives:**

We exposed neonatal rats to daily doses of diazinon or parathion on postnatal days (PND)1–4 and evaluated 5HT receptors and the 5HT transporter in brainstem and forebrain on PND5, focusing on doses of each agent below the maximum tolerated dose and spanning the threshold for cholinesterase inhibition: 0.5, 1, or 2 mg/kg for diazinon, and 0.02, 0.05, and 0.1 mg/kg for parathion.

**Results:**

Diazinon evoked up-regulation of 5HT_1A_ and 5HT_2_ receptor expression even at doses devoid of effects on cholinesterase activity, a pattern similar to that seen earlier for another organophosphate, chlorpyrifos. In contrast, parathion decreased 5HT_1A_ receptors, again at doses below those required for effects on cholinesterase. The two agents also differed in their effects on the 5HT transporter. Diazinon evoked a decrease in the brainstem and an increase in the forebrain, again similar to that seen for chlorpyrifos; this pattern is typical of damage of nerve terminals and reactive sprouting. Parathion had smaller, nonsignificant effects.

**Conclusions:**

Our results buttress the idea that, in the developing brain, the various organophosphates target specific neurotransmitter systems differently from each other and without the requirement for cholinesterase inhibition, their supposed common mechanism of action.

Organophosphates are undergoing increasing restrictions on their home use in the United States (U.S. Environmental Protection Agency 2000, 2002), but nonetheless they still account for > 50% of all insecticide use (Casida and Quistad 2004). One of the major concerns for human health is the propensity of these agents to produce developmental neurotoxicity, even when exposures are too low to elicit signs of systemic intoxication (Landrigan 2001; Landrigan et al. 1999; May 2000; Physicians for Social Responsibility 1995; Pope 1999; Slotkin 1999, 2004; Weiss et al. 2004). In that regard, chlorpyrifos has been the most studied organophosphate, and it is now clear that the original view of its mechanism of action—cholinesterase inhibition via its active metabolite, chlorpyrifos oxon—is insufficient to explain its ability to damage the developing brain. In fact, multiple mechanisms target neural cell replication and differentiation, axonogenesis and synaptogenesis, and the development and programming of synaptic activity, culminating in behavioral deficits (Barone et al. 2000; Casida and Quistad 2004; Gupta 2004; Pope 1999; Qiao et al. 2002, 2003; Yanai et al. 2002). There is an important corollary of the compound mechanisms for disruption of brain development: Whereas all organophosphates share actions directed toward cholinesterase, they may differ substantially in many of their noncholinesterase effects, such as actions directed toward oxidative stress, cell signaling, expression and function of nuclear transcription factors, and cell replication and differentiation (Gupta 2004; Pope 1999; Slotkin 1999, 2004, 2005), even if some of those additional mechanisms are shared by various organophosphates (Abu-Qare and Abou-Donia 2001; Morale et al. 1998; Pope 1999; Qiao et al. 2001; Slotkin 1999, 2004; Slotkin et al. 2006; Whyatt et al. 2002).

In a recent study (Slotkin et al. 2006), we compared the dose–effect relationships for systemic toxicity and developmental neurotoxicity for chlorpyrifos, diazinon, and parathion. Although parathion exhibited the highest systemic toxicity, it was actually less neurotoxic toward neurite formation and development of cholinergic projections, whereas diazinon and chlorpyrifos were less systemically toxic and more neurotoxic. In the present study we extended this approach to the evaluation of serotonergic (5HT) systems in the neonatal rat brain. Studies with chlorpyrifos show that 5HT systems are among the most sensitive to developmental disruption, with adverse effects detectable even when exposures lie below the threshold for inhibition of cholinesterase (Aldridge et al. 2003, 2004, 2005a, 2005b, 2005c; Qiao et al. 2002; Raines et al. 2001). Targeting of 5HT function is critical for three distinct reasons. First, 5HT is a morphogen in the developing mammalian central nervous system; perturbations of this system lead to errors in the architectural assembly of the brain (Hamon et al. 1989; Whitaker-Azmitia 1991, 2001). Second, disruption or enhancement of 5HT synaptic communication in early development permanently “programs” future 5HT function, so even greater neurobehavioral anomalies emerge later (Aldridge et al. 2004, 2005a, 2005b; Kusserow et al. 2004). Third, unlike the adverse effects on cholinergic systems, which typically involve cognitive deficits, alterations in 5HT function elicit changes in affective states, appetite, and sleep patterns (Aldridge et al. 2005a; Nemeroff 1998; Nutt 2002; Risch and Nemeroff 1991), thus expanding the scope of behavioral end points that need to be considered after early organophosphate exposure (Aldridge et al. 2005a). In the present study, we evaluated the immediate effects of neonatal treatment with doses of diazinon and parathion below the maximum tolerated dose (Slotkin et al. 2006) and spanning the threshold for barely detectable cholinesterase inhibition. Measurements were conducted for three 5HT synaptic proteins known to be affected by developmental exposure to chlorpyrifos (Aldridge et al. 2003, 2004; Raines et al. 2001; Slotkin and Seidler 2005): 5HT_1A_ and 5HT_2_ receptors and the presynaptic 5HT transporter. The two receptors converge on common end points in 5HT cell signaling (Barnes and Sharp 1999; Morin et al. 1992; Rovescalli et al. 1993) and are key players in 5HT-related mental disorders (Arango et al. 2001; Fujita et al. 2000; Yatham et al. 1999, 2000); the 5HT transporter regulates the synaptic concentration of 5HT and is the major target for antidepressant drugs (Maes and Meltzer 1995; Nemeroff 1998; Nutt 2002). Evaluations were conducted in the forebrain, which contains a high concentration of 5HT projections, and in the brainstem, which contains the corresponding cell bodies.

## Materials and Methods

### Animal treatments

All experiments were carried out humanely and with regard for alleviation of suffering, with protocols approved by the Institutional Animal Care and Use Committee and in accordance with all federal and state guidelines. Timed-pregnant Sprague-Dawley rats (Charles River, Raleigh, NC) were housed in breeding cages, with a 12-hr light/dark cycle and free access to food and water. On the day of birth, all pups were randomized and redistributed to the dams with a litter size of 9–10 to maintain a standard nutritional status. Because of their poor water solubility, diazinon and parathion (both from Chem Service, West Chester, PA) were dissolved in dimethylsulfoxide (DMSO) to provide consistent absorption (Whitney et al. 1995) and were injected subcutaneously in a volume of 1 mL/kg once daily on postnatal days (PND)1–4; control animals received equivalent injections of DMSO vehicle, which does not itself produce developmental neurotoxicity (Song et al. 1998; Whitney et al. 1995). Doses were chosen to lie below the threshold for signs of systemic toxicity as evidenced by impaired viability or reduced weight gain (Slotkin et al. 2006): 0.5, 1, and 2 mg/kg for diazinon, and 0.02, 0.05, and 0.1 mg/kg of parathion. The highest dose of each agent represents the maximum tolerated dose (Slotkin et al. 2006). On PND5, one male and one female pup were selected from each of at least six litters in each treatment group and were used for neurochemical evaluations. Animals were decapitated, the cerebellum was removed, and the brainstem and forebrain were separated by a cut made rostral to the thalamus. Tissues were weighed and flash frozen in liquid nitrogen and maintained at −45°C until analyzed. For a supplemental study determining the degree of cholinesterase inhibition immediately after treatment, additional animals were used to obtain samples 2 hr after the last injection of 2 mg/kg of diazinon or vehicle on PND4.

### Assays

Assays were conducted on each individual tissue, so that each determination represented a value from the corresponding brain region of one animal. Each tissue was thawed and homogenized (Polytron; Brinkmann Instruments, Westbury, NY) in ice-cold 50 mM Tris (pH 7.4), and aliquots of the homogenate were withdrawn for measurement of total protein (Smith et al. 1985) and cholinesterase activity (Ellman et al. 1961). For the latter, the homogenate was diluted in 0.5% Triton X-100, 0.1 M Na_2_HPO_4_/KH_2_PO_4_ (pH 8), and left on ice for 15 min to allow the Triton X-100 to solubilize membrane-associated cholinesterase. Homogenates were sedimented at 40,000 × *g* for 15 min, and aliquots of the supernatant solution were added to final concentrations of 0.5 mM acetylthiocholine iodide and 0.33 mM 5,5′-dithiobis(2-nitro-benzoic acid) in the same buffer without Triton (all reagents from Sigma Chemical Co., St. Louis, MO). Assays were incubated at room temperature for 4, 8, 12, 16, and 20 min, and the enzyme activity was assessed from the linear portion of the time course, reading the absorbance at 415 nm. The assay was standardized using mercaptoethanol standards and calculated relative to total protein.

The remaining homogenate was sedimented at 40,000 × *g* for 15 min; the resultant pellet was then washed by resuspension (Polytron) in homogenization buffer followed by resedimentation, and was then dispersed with a homogenizer (smooth glass fitted with Teflon pestle) in 50 mM Tris buffer (pH 7.4). An aliquot was withdrawn for the determination of membrane protein (Smith et al. 1985). Two radioligands were used to determine 5HT receptor binding (Xu et al. 2002): 1 nM [^3^H]8-hydroxy-2-(di-n-propylamino)tetralin (specific activity, 135 Ci/mmol; PerkinElmer Life Sciences, Boston, MA) for 5HT_1A_ receptors (Park et al. 1999; Stockmeier et al. 1998), and 0.4 nM [^3^H]ketanserin (specific activity, 63Ci/mmol; PerkinElmer) for 5HT_2_ receptors (Leysen et al. 1982; Park et al. 1999). For 5HT_1A_ receptors, incubations lasted 30 min at 25°C in a buffer consisting of 50 mM Tris (pH 8), 2 mM MgCl_2_, and 2 mM sodium ascorbate; 100 μM 5HT (Sigma) was used to displace specific binding. For 5HT_2_ receptors, incubations lasted 15 min at 37°C in 50 mM Tris (pH 7.4), and specific binding was displaced with 10 μM methylsergide (Sandoz Pharmaceuticals, E. Hanover, NJ). Incubations were stopped by the addition of a large excess of ice-cold buffer, and the labeled membranes were trapped by rapid vacuum filtration onto glass fiber filters that were presoaked in 0.15% polyethyleneimine (Sigma). The filters were then washed repeatedly and radiolabel was determined. For binding to the presynaptic 5HT transporter (Moret and Briley 1991; Slotkin et al. 1997, 1999, 2000; Xu et al. 2001), the membrane suspension was incubated with 85 pM [^3^H]paroxetine (specific activity 19.4 Ci/mmol; PerkinElmer) with or without addition of 100 μM 5HT to displace specific binding, and incubations lasted 120 min at 20°C. Binding was calculated relative to membrane protein.

### Data analysis

Data were compiled as means ± SEs. Because we evaluated multiple neurochemical variables that were all related to 5HT synapses, the initial comparison was conducted by a global analysis of variance (ANOVA; data log-transformed because of heterogeneous variance between regions and measures) incorporating all the variables and measurements in order to avoid an increased probability of type 1 errors that might otherwise result from multiple tests of the same data set: treatment, sex, region, and the three measures (5HT_1A_ receptors, 5HT_2_ receptors, 5HT transporter; repeated measure). Similarly, for cholinesterase activity, the initial ANOVA examined treatment, sex, and region. Where we identified interactions of treatment with the other variables, data were then subdivided for lower-order ANOVAs, followed by Fisher’s protected least significant difference test to evaluate individual treatments that differed from the corresponding control. However, where interactions are not significant, we report only the main treatment effects. Significance was assumed at the level of *p* < 0.05. For convenience, some of the results are presented as the percent change from control values, but statistical comparisons were conducted only on the original data. For reference, the corresponding control values are shown in Table 1.

## Results

**Figure 1:**
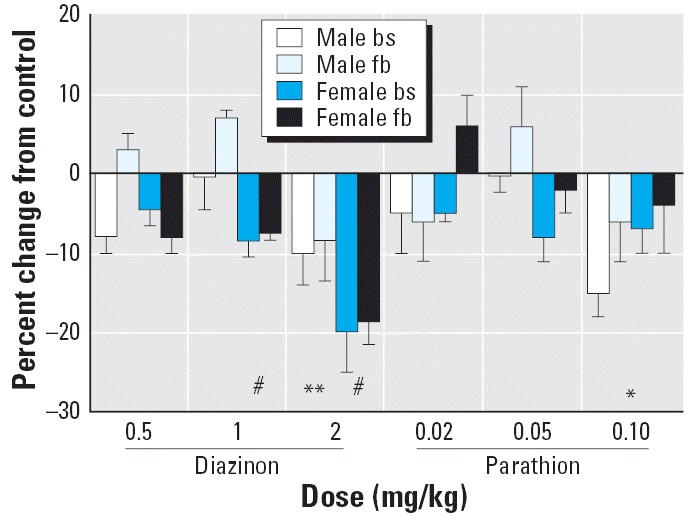
Cholinesterase activity on PND5, 24 hr after the last dose of diazinon or parathion. Abbreviations: bs, brainstem; fb, forebrain. Data are presented as the percentage change from control values (Table 1). ANOVA across all contributing variables indicates a main treatment effect (*p* < 0.0001) and an interaction of treatment × sex (*p* < 0.003); treatment effects were significant in both males (*p* < 0.0009) and females (*p* < 0.0001). *Significantly different from control (main effect without a treatment × sex interaction). **Significantly different from control males. #Significantly different from control females.

The doses we chose for daily treatment of neonatal rats with diazinon or parathion on PND1–4 spanned the threshold for significant inhibition of cholinesterase in the brainstem and forebrain (Figure 1). Twenty-four hours after the final injection, there was no significant inhibition in either the brainstem or forebrain at the lowest dose of diazinon (0.5 mg/kg). At 1 mg/kg, there was a barely detectable (< 10%) inhibition that achieved statistical significance in females but not in males, and at 2 mg/kg both sexes showed detectable inhibition. At the highest dose, cholinesterase was still inhibited only about 10% in males and 20% in females. With parathion treatment, neither 0.02 nor 0.05 mg/kg evoked significant cholinesterase inhibition. Raising the dose to 0.1 mg/kg produced statistically significant inhibition that was still only 5–15%.

**Figure 2:**
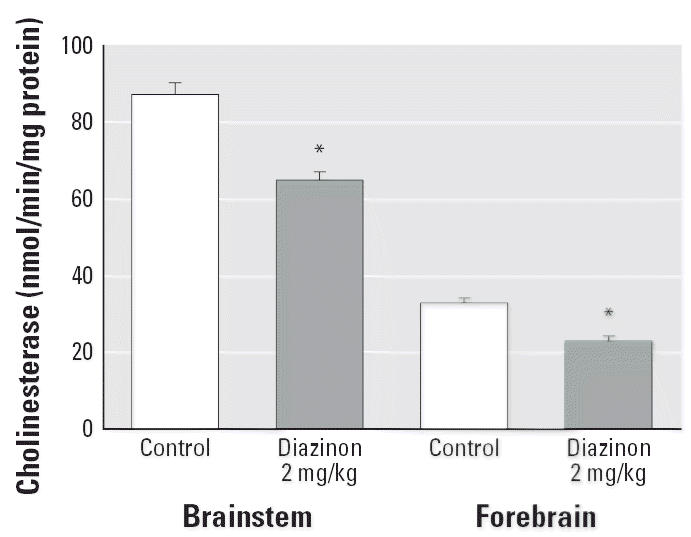
Cholinesterase activity on PND4, 2 hr after the last dose of 2 mg/kg diazinon. ANOVA across all contributing variables indicates a significant main treatment effect (*p* < 0.0001); results for males and females were combined because of the absence of a treatment × sex interaction. *Significantly different from control.

Cholinesterase activity recovers much more quickly in developing animals than in adults because of the rapid, growth-associated synthesis of new cholinesterase molecules (Chakraborti et al. 1993; Lassiter et al. 1998). Accordingly, we performed an additional experiment in which cholinesterase was measured 2 hr after the final injection of the highest dose of diazinon (Figure 2), a time point corresponding to the peak of inhibition (Dam et al. 2000). Under these conditions, there was somewhat more inhibition (25–30%) than that seen at the 24-hr time point, but the amount still was far less than the 70% inhibition threshold for signs of cholinergic hyperstimulation and systemic toxicity (Clegg and van Gemert 1999).

Both diazinon and parathion elicited significant effects on 5HT receptors and the 5HT transporter. Across all three measures and all contributing variables, global ANOVA indicated a significant main treatment effect (*p* < 0.0001) that reflected alterations for 0.5 or 1 mg/kg of diazinon (*p* < 0.0004 and *p* < 0.0006, respectively), and for 0.1 mg/kg of parathion (*p* < 0.02). However, the treatment effect also depended both on sex and region and on the specific measurement being made (treatment × sex × region × measure, *p* < 0.006). Accordingly, we subdivided the results for the three different proteins (5HT_1A_ receptor, 5HT_2_ receptor, 5HT transporter) and reexamined treatment effects across region and sex for each one separately.

**Figure 3:**
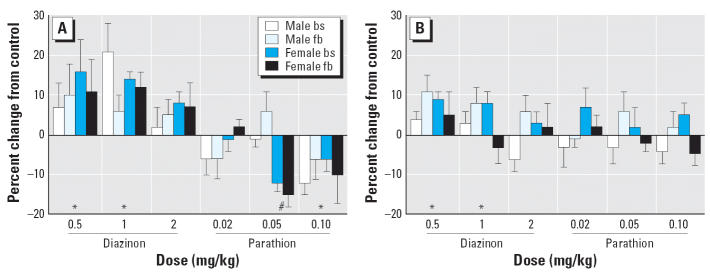
Effects of PND1–4 diazinon or parathion treatment on 5HT_1A_ receptors (*A*) and 5HT_2_ receptors (*B*).Abbreviations: bs, brainstem; fb, forebrain. Data are presented as the percentage change from control values (Table 1). For 5HT_1A_ receptors, ANOVA across all contributing variables indicates a main treatment effect (*p* < 0.0001) and a treatment × sex interaction (*p* < 0.02), with significant treatment effects for both males and females (*p* < 0.0001 for each). For 5HT_2_ receptors, there was only a main treatment effect (*p* < 0.03), so lower order tests for each sex were not conducted. *Significantly different from control (main effect without a treatment × sex interaction). #Significantly different from control females.

Neonatal treatment with diazinon or parathion produced significant changes in 5HT_1A_ receptor binding, reflecting a main treatment effect and a treatment × sex interaction, with significant effects for both males and females (Figure 3A). For diazinon, the two lower doses, 0.5 and 1 mg/kg, showed significant overall elevations without distinction between brainstem and forebrain and without preferential effects for males compared with females. Raising the dose to 2 mg/kg diminished the response so that it became non-significant. We observed a different pattern for parathion, which caused a significant decrease instead of increasing 5HT_1A_ receptor binding. Although there was no effect at the lowest dose of parathion, the intermediate dose elicited a significant reduction in females and the highest dose evoked a reduction in both sexes. The effects on 5HT_2_ receptors were also statistically significant but generally of smaller magnitude than those seen for the 5HT_1A_ sub-type (Figure 3B). In this case, there was only a main treatment effect, so no lower-order tests were run for separate regions or sexes. Again, the two lower doses of diazinon elicited a statistically significant increase in 5HT_2_ receptor binding, whereas the highest dose did not. Parathion did not have significant effects at any dose.

**Figure 4:**
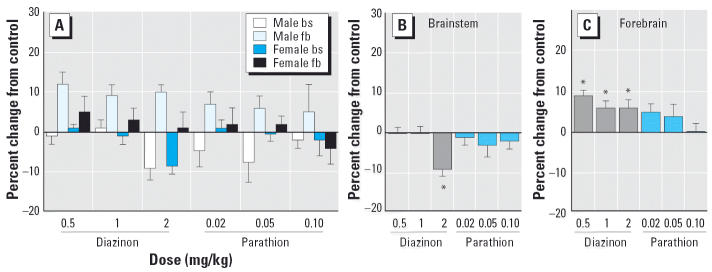
Effects of PND1–4 diazinon or parathion treatment on 5HT transporter binding (*A*). Abbreviations: bs, brainstem; fb, forebrain. Data are presented as the percentage change from control values (Table 1). ANOVA across all contributing variables indicates a significant treatment × region interaction (*p* < 0.02), with significant differences detected in both the brainstem (*p* < 0.02) and forebrain (*p* < 0.05). Accordingly, results were subdivided into brainstem (*B*) and forebrain (*C*), but combined for males and females because of the absence of a treatment × sex interaction. *Significantly different from control.

For the 5HT transporter, there was a significant treatment × region interaction (Figure 4A), necessitating separate examination of the effects in the brainstem (Figure 4B) and forebrain (Figure 4C). Because there was no treatment × sex interaction, we show the results for males and females combined. In the brainstem, diazinon reduced transporter expression but only at the highest dose; parathion had no effect. In the forebrain, diazinon increased the values significantly, whereas parathion was less effective (not statistically significant).

## Discussion

In our earlier work with chlorpyrifos, we found that exposure in the early postnatal period elicited an immediate increase in 5HT receptor expression (Aldridge et al. 2003). Because these effects occurred during the critical period in which 5HT synaptic development and function are programmed (Kusserow et al. 2004), much larger alterations appeared in adolescence and adulthood, accompanied by defective 5HT synaptic transmission and behavioral deficits (Aldridge et al. 2004, 2005a, 2005b). In the present study, diazinon elicited a similar up-regulation of 5HT receptors, demonstrating that the effects are not unique to chlorpyrifos. Nevertheless, this is not a property shared by all organophosphates; parathion failed to increase 5HT receptor expression, and instead evoked a decrease with exposures approaching the maximum tolerated dose (Slotkin et al. 2006). This dichotomy argues strongly against a specific role of cholinesterase inhibition as a common mechanism underlying the developmental neurotoxicity of organophosphates. Indeed, just as found previously for chlorpyrifos (Aldridge et al. 2003; Qiao et al. 2002), the effects of diazinon on 5HT systems were apparent at exposures below the threshold for cholinesterase inhibition. In fact, raising the dose above that threshold actually diminished the 5HT receptor up-regulation, suggesting that there is a nonmonotonic dose–effect relationship, with promotional actions at low doses offset by inhibitory ones as the exposure is raised above the threshold for cholinesterase-related systemic toxicity. Again, similar findings of a biphasic response have been reported for neurodevelopmental effects of chlorpyrifos (Levin et al. 2002; Qiao et al. 2002). The effects of diazinon on 5HT receptors in the present study thus resemble those of chlorpyrifos in direction and magnitude (Aldridge et al. 2003). Future studies should address whether, like chlorpyrifos, these events presage much larger changes that emerge in adolescence and adulthood (Aldridge et al. 2004, 2005a, 2005b). In contrast, based on the opposite effect of parathion on 5HT receptors observed in the present study, we would predict an entirely different long-term outcome for exposure to that organophosphate.

In contrast to its promotional effects on 5HT receptors, diazinon had differential effects on expression of the 5HT transporter in the two brain regions. In the brainstem, diazinon reduced transporter binding values, but only when the dose was raised above the threshold for cholinesterase inhibition; in contrast, values were elevated in the forebrain even at low doses. Again, these are generally similar to the results from our earlier work with chlorpyrifos (Raines et al. 2001), although the effects of diazinon are more robust. With cholinergic agonists such as nicotine, damage to neuronal projections in the forebrain results in reactive sprouting at the cell body (Xu et al. 2001); if diazinon were similar, we would have seen reductions in 5HT transporter expression in the forebrain and increases in the brainstem, the opposite of what we actually obtained. On the other hand, neurotoxicants that directly target developing monoamine terminals tend to produce sprouting further along the axon, thus producing the corresponding increment in the forebrain, which contains the terminal zone (Blue and Molliver 1987), just as seen here. Again, this argues strongly for effects of diazinon (and potentially of chlorpyrifos as well) on developing 5HT projections that are independent of effects on cholinesterase or cholinergic systems. Notably, parathion was far less effective (no significant changes) for such actions in either brain region, reinforcing the fact that the various organophosphates can elicit different outcomes. Chlorpyrifos has already been shown to have differential effects on dendritic versus axonal outgrowth (Howard et al. 2005); therefore, it is likely that diazinon has similarly disparate effects on different types of neuritic projections.

The case of parathion is particularly intriguing, especially given its much greater systemic toxicity, which has a maximum tolerated dose over an order of magnitude lower than that of chlorpyrifos or diazinon in neonatal rats (Liu et al. 1999; Slotkin et al. 2006). We previously found that parathion, at its maximum tolerated dose, did not compromise the development of neuritic projections or emergence of the cholinergic phenotype, whereas chlorpyrifos did (Jameson et al. 2006; Slotkin et al. 2006). In the present study, parathion, even at a dose below the threshold for cholinesterase inhibition, had a deleterious effect on the development of 5HT_1A_ receptors, a unique effect not shared by diazinon or chlorpyrifos (Aldridge et al. 2003). Accordingly, parathion “spares” developing neurites and cholinergic projections because its greater systemic toxicity limits the dose that can be given, rather than because it is inherently less neurotoxic (Slotkin et al. 2006). However, in the case of 5HT systems, it appears to have additional, direct effects that are independent of cholinesterase inhibition and that can be evoked below the maximum tolerated dose. Again, it will be useful to pursue the long-term neurochemical and behavioral consequences of these actions.

## Conclusion

Our results bolster the idea that, in the developing brain, the various organophosphates target specific neurotransmitter systems differently and without the requirement for cholinesterase inhibition, their supposed common mechanism of action (Mileson et al. 1998). In fact, the 5HT system is especially vulnerable to disruption by diazinon, chlorpyrifos, and parathion, with parathion showing a distinctly different spectrum of actions from the other two agents. The fact that alterations in neurodevelopment occur with organo-phosphate exposures below the threshold for cholinesterase inhibition reinforces the inadequacy of this biomarker for assessing exposure or outcome related to developmental neurotoxicity. Finally, the differential effects of the various organophosphates raise the intriguing possibility that safer compounds could be engineered that avoid the critical mechanisms evoking developmental neurotoxicity.

## Figures and Tables

**Table 1:** Control values.

	Brainstem		Forebrain	
	Male	Female	Male	Female
5HT_1A_ receptors (fmol/mg membrane protein)	62 ± 1	64 ± 2	49 ± 2	53 ± 1
5HT_2_ receptors (fmol/mg membrane protein)	33 ± 1	32 ± 1	42 ± 1	45 ± 1
5HT transporter (fmol/mg membrane protein)	390 ± 8	392 ± 6	174 ± 5	184 ± 4
Cholinesterase (nmol/min/mg total protein)	79 ± 3	79 ± 2	31 ± 1	32 ± 1
